# Quantification of Trunk Postural Stability Using Convex Polyhedron of the Time-Series Accelerometer Data

**DOI:** 10.1155/2016/1621562

**Published:** 2016-04-20

**Authors:** Roman Melecky, Vladimir Socha, Patrik Kutilek, Lenka Hanakova, Peter Takac, Jakub Schlenker, Zdenek Svoboda

**Affiliations:** ^1^Faculty of Biomedical Engineering, Czech Technical University in Prague, 272 01 Kladno, Czech Republic; ^2^Department of Rehabilitation and Spa Medicine, Faculty of Medicine, P. J. Šafárik University in Košice, 040 01 Košice, Slovakia; ^3^Palacky University of Olomouc, Faculty of Physical Culture, 771 11 Olomouc, Czech Republic

## Abstract

Techniques to quantify postural stability usually rely on the evaluation of only two variables, that is, two coordinates of COP. However, by using three variables, that is, three components of acceleration vector, it is possible to describe human movement more precisely. For this purpose, a single three-axis accelerometer was used, making it possible to evaluate 3D movement by use of a novel method, convex polyhedron (CP), together with a traditional method, based on area of the confidence ellipse (ACE). Ten patients (Pts) with cerebellar ataxia and eleven healthy individuals of control group (CG) participated in the study. The results show a significant increase of volume of the CP (CPV) in Pts or CG standing on foam surface with eyes open (EO) and eyes closed (EC) after the EC phase. Significant difference between Pts and CG was found in all cases as well. Correlation coefficient indicates strong correlation between the CPV and ACE in most cases of patient examinations, thus confirming the possibility of quantification of postural instability by the introduced method of CPV.

## 1. Introduction

Postural stability of the body segments during standing, especially trunk stability, can be negatively influenced by many diseases of the nervous or musculoskeletal system [1]. Patients with these deficits often show instability during stance tasks [2]. Thus, the trunk accelerations during stance can be quantitative indicators of impaired balance control in individuals with neurological disorders [3]. Hence, the evaluation of accelerations suits the needs of clinical practice since they reflect the changes in position as well as the intensity and magnitude of tremor. The biomedical community is currently starting to use the triaxial inertial measurement units (IMU) for high-accuracy measurement of human body segment movements (accelerations and orientations) instead of commonly used force (posturography) platforms used to study the center of pressure (CoP) movements of whole body [4]. Assessment of trunk movements using IMU may yield clearer insights into balance deficits and provide a considerably cheaper and better diagnostic tool than more traditional, previously documented measures. The IMUs were placed on spinous processes of T1 and/or S1 for measuring the motion of trunk and pelvis during quiet standing. Although the IMU can measure three angles and three accelerations, the techniques to quantify segment movements using only one or two measured quantities were introduced in clinical practice [4]. Thus, in clinical practice, one of the greatest advantages of the IMU compared to traditional posturography platforms, which allow for measuring only the 2D movements in transversal plane, is not used. The reason for this is that the application of the IMU for trunk acceleration measurement during stance is relatively new and the IMU has not been previously used to study the range postural balance problems and patients with specific types of diseases. Thus, the posturography platforms are always the main tool for the study of body movement of the patients, for instance, suffering from cerebellar diseases [5]. Therefore, the first objective of this paper is to test the application of the IMU in an area where IMUs have not been used before and where IMUs will replace conventional posturography platforms.

Since the design is intended to identify connections between trunk movement in space and neurological disorders, it is used to diagnose cerebellar disease characterized by tremor or sway. Cerebellar ataxia can arise as a result of many diseases and present itself with symptoms of coordination, balance, gait, and extremity movements difficulties. Lesions to the cerebellum can cause dyssynergia, dysmetria, dysdiadochokinesia, dysarthria, ataxia of stance and gait, and so forth; see [6, 7]. Deficits can be observed on movements on the same side of the body as the lesion [6]. Clinicians often use motor tasks in order to verify the signs of ataxia [7]. Patients with a cerebellar ataxia diagnosis are interesting not only because of their impaired postural stability but also because there is no effective causal pharmacotherapy. Therefore, it is necessary to look for methods which can enhance the accuracy of evaluation procedures of patients' postural stability during the treatment.

The second objective of the paper is to design and test a new method of quantitative evaluation of 2D data set for the 3D trunk movement measured by IMU. Traditional, more complex methods for processing the measured data and assessing the postural instability, using at least two measured variables, are methods based on the 2D convex hull, 2D confidence ellipse, or length of trajectory obtained by plotting two variables versus each other [8–10]. Usually, these methods are used to evaluate 2D data set from posturography platforms [6]. However, there are limitations of these traditional solutions to quantify postural stability which were originally used to evaluate 2D data set from the posturography platforms and which are now being used to evaluate data from the IMUs. As mentioned before, the limitation is that these methods are based solely on the evaluation of only two variables, each in one of the two human body planes/axes. This can lead to a loss of important information about physical activity, specifically the third physical quantity (i.e., acceleration) of 3D movement. However, we can also model the distribution of the measured 3D data (i.e., three orthogonal accelerations) of human body segment instead of the analysis of 2D data.

Therefore, this study is aimed at introducing a novel method used in the identification of pathological balance control using IMU to measure accelerations as well as the convex polyhedron of plotting three accelerations versus each other (the evaluation of 3D data set of superior-inferior acceleration, mediolateral acceleration, and anterior-posterior acceleration of body segments). It follows practice consisting in the assessment of the convex hull of only two variables (specifically angles) measured by IMU [11]. The convex hull area has already been used in clinical practice to study postural balance problems, but the concept of convex polyhedron volume (CPV) has never been used before in clinical practice to study postural balance problems by three accelerations [8]. The choice for this novel design came from the ability of a single variable which defines the shape of the convex polyhedron used to describe changes in three accelerations, considering wide availability of new cheaper triaxial IMUs (ordinary cell phones or watches) [12, 13]. The applicability of the convex polyhedron variable describing the body segment movement foreshadows the immense potential of a simple and inexpensive IMU capable of direct evaluation of complex 3D movement (i.e., 3D acceleration) as a whole. Moreover, the combinations of superior-inferior, mediolateral, and anterior-posterior acceleration measurements during specific balance tasks could identify new and specific differences in balance control of patients compared to healthy subjects. The final significant reason for the measurement and evaluation of all three accelerations, instead of only two, is that the calibration of the cheap triaxial IMU may not be accurate, and thus the measurement of all three accelerations minimalizes the loss of information about the 3D movement as a whole. The inclusion of all three accelerations allows us to minimalize the influence of mispositioning the IMU on a body segment. The claim follows the general assumption that acceleration vector in 3D space is defined by the three segments of the vector, while these segments are typically considered in accordance with the Earth's coordinate system axes or anatomical axes of a particular body part. If the sensor is misplaced on the body part or miscalibrated as for the coordinate system, the measured segments of acceleration in their respective axes will be inaccurate. If we choose to examine 3D movement by using only two segments of acceleration, a piece of information may be lost, which is never the case if all three segments are observed. The inclusion of all three accelerations allows us to minimalize the influence of mispositioning of the IMU on a body segment.

The method of recording and processing 3D data thus offers the possibility to measure stability in smaller medical centers or at home by using IMU implemented in, for example, a mobile phone instead of the traditional and spacious posturography platforms. There is also a possibility for patients to perform the therapy, examination, or training at home. The method also eliminates the risk of mispositioning the sensor and loss of vertical movement data and at the same time presents patients and medical staff with an easy-to-interpret 3D data processing method. Therefore, the focus of this work is to identify suitability of the convex polyhedron and IMU data for clinical application.

## 2. Methods and Materials

### 2.1. Participants

In order to test the new methods, it is necessary to compare healthy subjects without any postural balance problems to participants who have postural balance problems. Ten volunteer patients (Pts) (six women and four men; age of 52.2 (SD 11.7) years) with degenerative cerebellar ataxia participated [7]. The patients were recruited from the Faculty Hospital Motol, Prague, Czech Republic. A board-certified neurologist had previously diagnosed progressive cerebellar disease. Diagnostic evaluation included a neurologic examination, laboratory blood tests, and a brain MRI. The patients were measured in the initial phase of the clinic's two-week rehabilitation program. Eleven healthy individuals of control group (CG) (five women and six men; age of 26.0 (SD 6.4) years) were also recruited for comparable analysis. Healthy subjects were recruited from the students/volunteers at Charles University in Prague. In the case of the CG, the diagnostic evaluation included a detailed disease history, a neurological examination, and a routine laboratory testing. The study was performed in accordance with the Helsinki Declaration. The study protocol was approved by the local Ethical Committee and the University Hospital Motol, and an informed consent was obtained from each subject. The subjects were chosen for measurement randomly and on different days.

### 2.2. Measurement Equipment and Test Procedure

#### 2.2.1. Measurement Equipment

**Figure 1:**
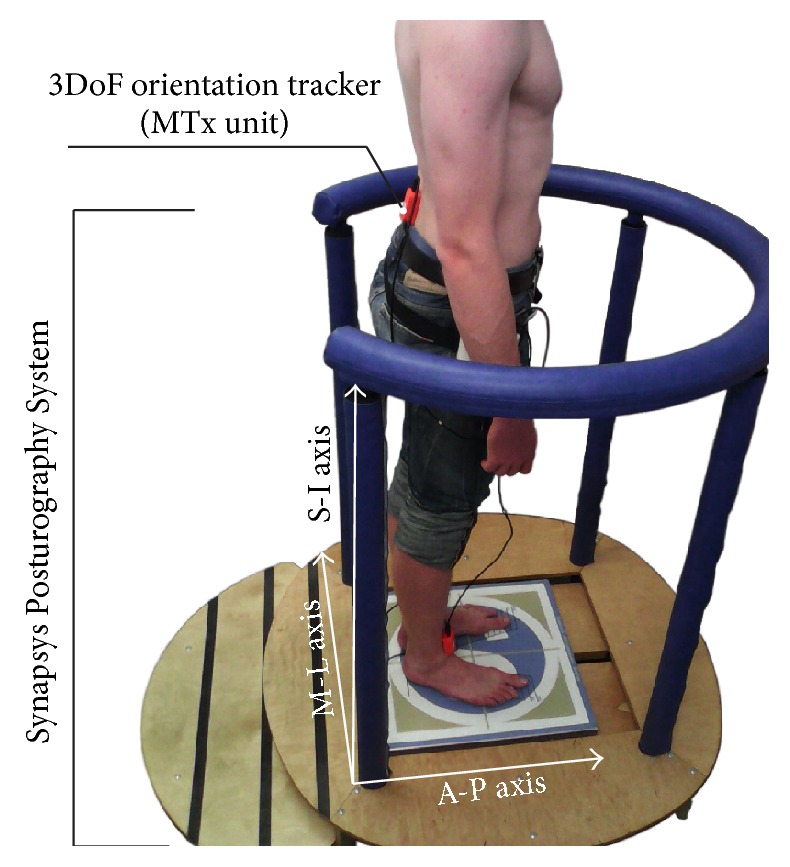
The MTx unit used to measure angles and accelerations of the trunk and the Synapsys Posturography System used to measure the COP displacements; S-I: superior-inferior axis, M-L: mediolateral axis, and A-P: anterior-posterior axis.

Xbus Master, a lightweight (330 g) and portable device using Motion Tracker Xsens (product abbreviation: MTx) units for orientation and acceleration measurement of body segments (see Figure 1), was used for measurements of trunk movements. MTx unit with an embedded accelerometer and gyroscope is accurate IMU measuring drift-free 3D orientation and 3D acceleration. The MTx unit was calibrated before each clinical examination by calibration measurement. The MTx unit was set up in a way that the one axis of the coordinate system of the MTx unit was parallel to the anterior-posterior axis, that is, symmetry axis of the fixed stationary platform of the Synapsys Posturography System on which the participants stood, and the other two axes were perpendicular to the anterior-posterior axis (i.e., symmetry axis of the platform) with respect to the direction of Earth's gravity; that is, superior-inferior axis was colinear with the direction of gravity. After calibration, the MTx unit was placed on patient's trunk according to [14, 15], at the level of the lower back (lumbar 2-3); see Figure 1.

The data sets comprised of the three Euler angles (roll (Φ), yaw (Ψ), and pitch (Θ)) as well as three orthogonal accelerations (*a*_*Sx*_, *a*_*Sy*_, *a*_*Sz*_) in the accelerometer coordinate system (i.e., three orthogonal components of acceleration direction corresponding to the three principal axes of the MTx unit accelerometer) were measured using an MTx unit placed on the subjects trunks while Pts and CG were performing a quiet stance on a fixed stationary platform of the Synapsys Posturography System [16, 17]. Conventions of Euler angles are described in [18]. The three accelerations measured by the accelerometer of MTx unit are described in detail in [19].

Also, measurement of the human body center of pressure (COP) displacement (i.e., postural sway) by force platform, Synapsys Posturography System, was performed to compare the data obtained by the traditional method with the data obtained by the IMU. The Synapsys Posturography System provides information about the area of the 95% confidence ellipse of COP excursions [20]. Comparison between COP characteristics and accelerometer data took place due to the fact that COP movement is given by COM (center of mass) movement, position of which follows the position of individual segments. A significant body segment is the trunk on which the accelerometer (i.e., IMU) is placed. Change in the position of the trunk, which is measured as acceleration using IMU, thus directly affects the position of COM and therefore also COP. These data can of course differ with respect to the influence of other segments on the COP position; it is however assumed that the trunk position (or its movement) has the most significant influence on the COP position change. It would be examined and verified whether there is indeed a correlation between the data from the IMU and data from the posturography platform data, which would suggest that the platform is fully replaceable by the IMU.

#### 2.2.2. Test Procedure

The body sway of each participant was measured by the Xsens system (Xsens Technologies B.V.) and Synapsys Posturography System (Synapsys Inc.) during quiet stance on a firm surface (FiS) and soft foam surface (FoS) with eyes open (EO) and eyes closed (EC) [21].

The sequence of the four measurement settings for each subject was as follows: EO FiS, EC FiS, EO FoS, and EC FoS. The order of the four settings was set and followed in all participants. The subject's bare feet were positioned next to each other, splayed at the angle of 30°, and arms were always in hanging position. The tasks included standing on both feet for at least 60 seconds [22]. Both systems recorded body activity at the same time; that is, data were recorded simultaneously by both systems. Time synchronization of the measured data (i.e., data from both systems) was achieved by controlling both systems and processing data on the same computer. The measurements usually lasted a few seconds longer, and the initial data have been cut off so that all data sets have a record length of 60 seconds. The data were recorded with a sample frequency of 100 Hz (for both systems). Kalman filter was implemented in the Xbus Kit system and the MT Manager software of the Xbus Kit system was used for data storage.

### 2.3. Data Processing Method

The three Euler angles and three accelerations in the accelerometer coordinate system are used to calculate the accelerations in the global reference system and then in the anatomical coordinate frame. The calculation is based on the rotational matrices. The first rotation matrix *R*_*GS*_ rotates an acceleration vector ${\vec{a}}_{S}={\left(\begin{array}{ccc} a_{Sx} & a_{Sy} & a_{Sz} \end{array}\right)}^{T}$ in the sensor coordinate system (*S*) to the global reference system (*G*):

(1) $$\begin{array}{c} {\vec{a}}_{G}=R_{GS}\cdot {\vec{a}}_{S}, \end{array}$$

where the matrix *R*_*GS*_ is interpreted in terms of Euler angles [23]:

(2) $$\begin{array}{c} R_{GS}=R_{\Psi }^{Z}\cdot R_{\Theta }^{Y}\cdot R_{\Phi }^{X}, \end{array}$$

where

(3) RΨZ=cos⁡Ψ−sin⁡Ψ0sin⁡Ψcos⁡Ψ0001,RΘY=cos⁡Θ0sin⁡Θ010−sin⁡Θ0cos⁡Θ,RΦX=1000cos⁡Φ−sin⁡Φ0sin⁡Φcos⁡Φ.

The acceleration vector ${\vec{a}}_{G}={\left(\begin{array}{ccc} a_{Gx} & a_{Gy} & a_{Gz} \end{array}\right)}^{T}$ in the global reference system is then rotated to the anatomical coordinate frame (*A*):

(4) $$\begin{array}{c} {\vec{a}}_{a}=R_{AG}\cdot {\vec{a}}_{G}, \end{array}$$

where second rotation matrix *R*_*AG*_ is

(5) $$\begin{array}{c} R_{AG}=R_{\Psi_{0}}^{Z}=\left[\begin{array}{ccc} \cos\Psi_{0} & -\sin\Psi_{0} & 0\\ \sin\Psi_{0} & \cos\Psi_{0} & 0\\ 0 & 0 & 1 \end{array}\right]. \end{array}$$

**Figure 2:**
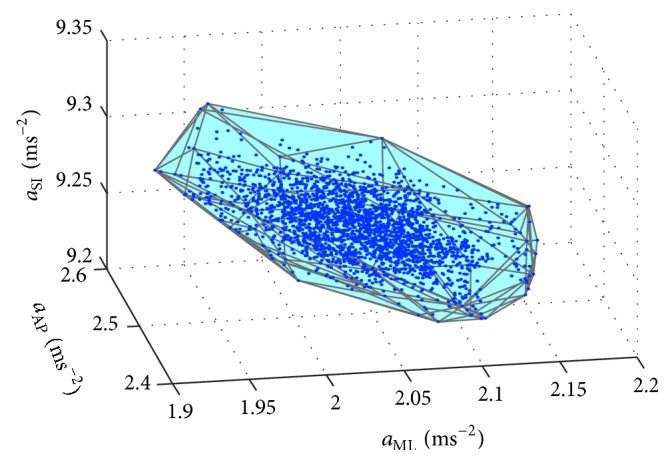
Example of a convex polyhedron obtained by plotting superior-inferior (SI), mediolateral (ML), and anterior-posterior (AP) accelerations versus each other.

The angle sin⁡Ψ_0_ is obtained during the calibration process of the MTx unit. The calculated acceleration vector ${\vec{a}}_{A}={\left(\begin{array}{ccc} a_{AP} & a_{ML} & a_{SI} \end{array}\right)}^{T}$ represents the superior-inferior acceleration (*a*_SI_), mediolateral acceleration (*a*_ML_), and anterior-posterior acceleration (*a*_AP_). The acceleration vectors, or in other words, time dependent data (*a*_SI_, *a*_ML_, *a*_AP_) are plotted as a 3D plot. The set of points is obtained by plotting the accelerations versus each other; see Figure 2. The time of measurement, that is, record length of the data set (60 s), and the sample frequency (100 Hz) affect the number of points in the set. Recording frequency must be sufficiently high to record also a short time and random displacements of the body segment, and no information about the range of motion during maintaining postural stability of stance is lost. Using a greater number of registered points of higher frequency, it is possible to record movement more accurately without losing information on certain phases of rapid movement that is registered through low frequency data collection. For instance, information loss could occur on tremor of segments caused by higher frequencies. Therefore, the data was collected at the frequency of 100 Hz using MT Manager Version 1.7.0, adjusted for human movements, as recommended by the manufacturer [24]. The choice of the sampling frequency of 100 Hz was made also by following previous studies which used the same system for motion tracking (see [25–29]).

The novel method for identification of pathological balance control is based on mathematical tools for static posturography [20, 30]. We can model the distribution of the measured data by 2D convex hull or 3D convex polyhedron (CP) [31]. A variable which can be used to describe the shape of the 2D convex hull or 3D convex polyhedron can be the area or volume. We used a method based on the description of the distribution of the measured data (i.e., *a*_SI_, *a*_ML_, and *a*_AP_) by CP. In mathematics, the convex hull of a set of points (SP) in the Euclidean space is the smallest convex set that contains SP [32]. The CP may be defined as the intersection of all convex sets containing SP or as the set of all convex combinations of points in SP [33]. The set of points is obtained by plotting three accelerations versus each other; see Figure 2. The CP of a set of points in 3D space is the smallest convex region enclosing all points in the set; see Figure 2. The number of points is determined by the time of measurement (60 s) and the sample frequency (100 Hz). A custom-designed MATLAB program based on the functions of the MATLAB software (MATLAB R2010b, Mathworks, Inc., Natick, MA, USA) was used to calculate the CP of the 3D plot. The convex hull computation in MATLAB uses the Delaunay triangulation [34]. Since there is no known method of calculating the CPV, we can use the equations used to calculate the volume of any polyhedron (PV) [35–37]:

(6) $$\begin{array}{c} PV=\frac{1}{3}\left|\;\overset{j}{\underset{i=1}{\sum }}P_{1i}\cdot N_{i}\cdot A_{i}\;\right|, \end{array}$$

where *A*_*i*_ is the surface area of a planar polygonal face *S*_*i*_:

(7) $$\begin{array}{c} A_{i}=\frac{1}{2}\left|\;N_{i}\cdot \overset{l}{\underset{k=1}{\sum }}\left(\;P_{ki}\times P_{\left(k+1\right)i}\;\right)\;\right|. \end{array}$$

Thus

(8) $$\begin{array}{c} PV=\frac{1}{6}\left|\;\overset{j}{\underset{i=1}{\sum }}\left(\;P_{1i}\cdot N_{i}\;\right)\cdot \left|\;N_{i}\cdot \overset{l}{\underset{k=1}{\sum }}\left(\;P_{ki}\times P_{\left(k+1\right)i}\;\right)\;\right|\;\right|, \end{array}$$

where *P*_1*i*_,…, *P*_*li*_ are the vertices of a planar polygonal face *S*_*i*_ oriented counterclockwise with respect to the outward pointing normal of planar polygonal face and *N*_*i*_ is a unit outward pointing vector normal to specific planar polygonal face *S*_*i*_:

(9) $$\begin{array}{c} N_{i}=\frac{\left(P_{2i}-P_{1i}\right)\times \left(P_{3i}-P_{1i}\right)}{\left|\left(P_{2i}-P_{1i}\right)\times \left(P_{3i}-P_{1i}\right)\right|}. \end{array}$$

To calculate the PV, the custom-designed MATLAB program was also used. Because the volume corresponds to the volume of CP of 3D plot obtained by plotting *a*_SI_, *a*_ML_, and *a*_AP_ versus each other, the physical unit of the volume is m^3^·s^−6^. Although the MTx unit also senses the gravitational acceleration, it is not necessary to subtract the gravitational acceleration because the method of calculating the PV uses only changes in the accelerations and the gravitational acceleration is constant and perpendicular to the horizontal plane of the Earth's surface.

The area of the 95% confidence ellipse (ACE) of COP excursions was used to compare the data obtained by the posturography system with data obtained by the IMU. The Synapsys Posturography System directly calculates the areas. Thus, it is not necessary to convert the measured data. The physical unit of the area is mm^2^.

### 2.4. Statistical Analysis

After calculating the CPV of each patient and healthy subject standing on a FiS and FoS with EO and EC, the Jarque-Bera test was used to test the normal distribution of calculated CPVs. The median (Mdn), minimum (Min), maximum (Max), first quartile (Q1), and third quartile (Q3) of the CPV were then used to compare the results. Also, the Wilcoxon signed rank test and Wilcoxon rank sum test were used to assess the significance of the differences between the measurements results, that is, to compare the different stance conditions and CG with Pts. The significance level was set at *p* < 0.05. Also, Spearman's rank correlation coefficient between the volume of convex polyhedron and the area of the confidence ellipse of COP excursions were calculated to study the differences between the data from IMU and the center of pressure data. The statistical analysis was performed using MATLAB software.

A comparison of the same age groups is not necessary, since studies show that parameters of the body sway of the healthy subjects within the age range between 20 and 60 years vary only slightly [38, 39]. Aoki et al. [38] found that there are insignificant differences in 10–60-year-old subjects in COP sway parameters (i.e., Romberg quotients). Also, a detailed analysis of age-related increase of CoP parameters by the polynomial type of regression showed that the gradual increase of body sway, that is, significant degradation of stability, characterized by increase of CoP oscillations, starts after the age of 60 [39].

## 3. Results

**Figure 3:**
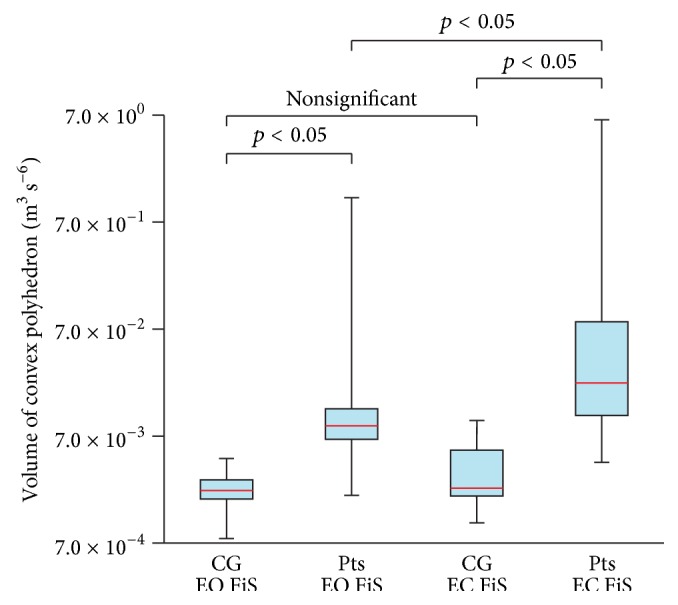
Comparison of the volume of convex polyhedron of control group (CG) and patients (Pts) standing on a firm surface (FiS) with eyes open (EO) and eyes closed (EC).

**Figure 4:**
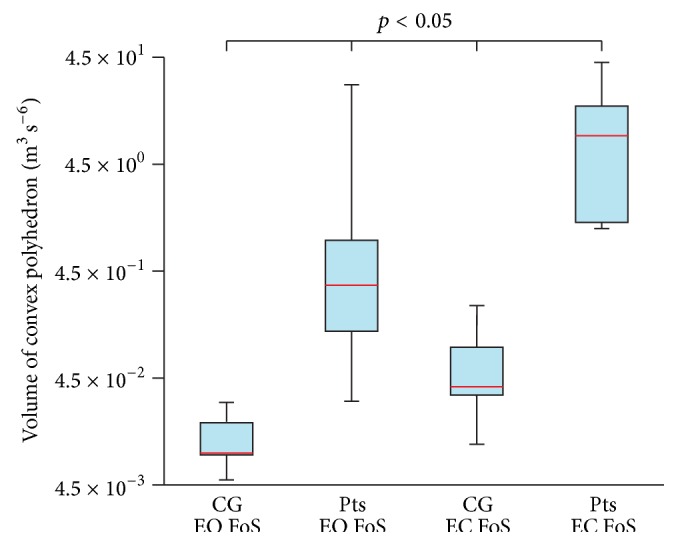
Comparison of the volume of convex polyhedron of control group (CG) and patients (Pts) standing on a foam surface (FoS) with eyes open (EO) and eyes closed (EC).

**Figure 5:**
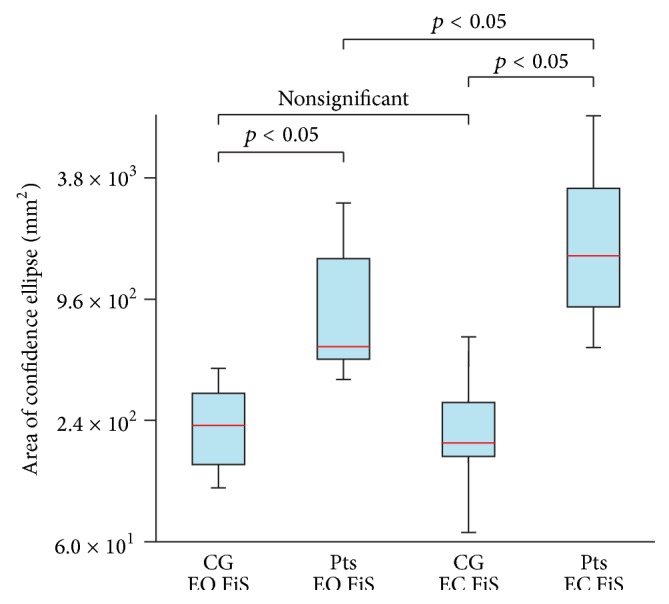
Comparison of the area of confidence ellipse of control group (CG) and patients (Pts) standing on a firm surface (FiS) with eyes open (EO) and eyes closed (EC).

**Figure 6:**
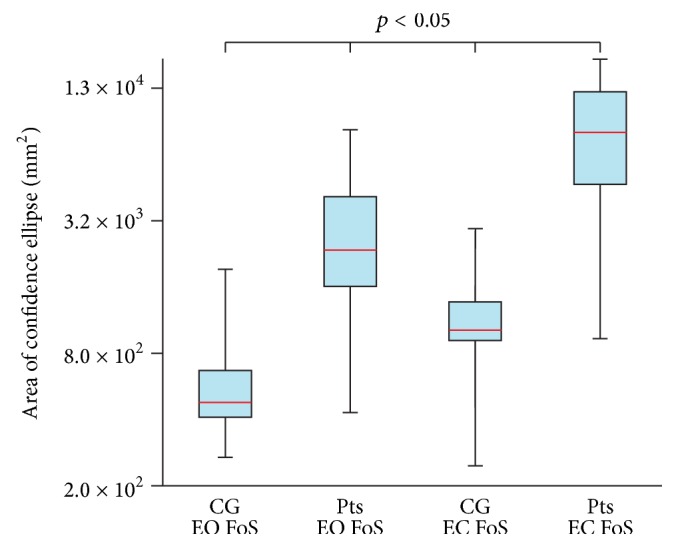
Comparison of the area of confidence ellipse of control group (CG) and patients (Pts) standing on a foam surface (FoS) with eyes open (EO) and eyes closed (EC).

The statistical data are used to illustrate the differences between the CPVs of Pts and CG; see Figures 3 and 4. Results obtained from the posturography platform are listed as well (see Figures 5 and 6) to provide for the evaluation of the data from IMU by comparing them with the data from the posturography platform. The following plots display the Min, Max, Mdn, Q1, and Q3 for the calculated CPVs and ACEs. Since some calculated values were not distributed normally, the Wilcoxon test was used to compare and analyze the data sets. In all cases of comparisons between the groups of data, the effect sizes ranged from moderate to large; that is, calculated values were greater than 0.5. G^*∗*^Power software (G^*∗*^Power 3.1.9., Universität Kiel, Germany) was used for the calculations.

### 3.1. Comparing Quiet Stance Trials

The comparison of CG on FiS with EO and CG on FiS with EC (*p* = 0.206) did not show any differences. Differences were found when comparing Pts on FiS with EO and Pts on FiS with EC (*p* = 0.014), CG on FoS with EO and CG on FoS with EC (*p* = 0.003), and Pts on FoS with EO and Pts on FoS with EC (*p* = 0.002). In the case of the CG or Pts with EO and EC standing on the FiS, the measured data show the slight increase of the median of the CPVs after the eyes closed phase (Figure 3). In the case of the CG or Pts with EO and EC standing on the FoS, the measured data show a significant increase of the median of the CPVs after the EC phase (Figure 4).

### 3.2. Comparing Patients and Healthy Subjects

Significant differences were found when comparing CG on FiS with EO and Pts on FiS with EO (*p* = 0.010), CG on FiS with EC and Pts on FiS with EC (*p* = 0.005), CG on FoS with EO and Pts on FoS with EO (*p* = 0.001), and CG on FoS with EC and Pts on FoS with EC (*p* = 0.001). In all cases, significant differences between the data for CG and Pts were observed.

The median of the CPVs in Pts standing on the FiS with EO is 4.0 times larger than the median of the CPVs in the CG standing on the FiS with EO. The median of the CPVs in Pts standing on the FiS with EC is 9.7 times larger than the median of the CPVs in the CG standing on the FiS with EC. The median of the CPVs in Pts standing on the FoS with EO is 37.2 times larger than the median of the CPVs in the CG standing on the FoS with EO. The median of the CPVs in Pts standing on the FoS with EC is 222.4 times larger than the median of the CPVs in the CG standing on the FoS with EC.

### 3.3. Correlation between the Data from IMU and the Center of Pressure Data

In the case of the CG with EO standing on the FiS and FoS, the Spearman rank correlation coefficient indicates a negligible correlation between the CPV and ACE. In the case of the CG with EC standing on the FiS and FoS, the correlation between the CPV and ACE is weak. In most cases, the patient examinations show strong correlation between the CPV and ACE; see Table 1. A moderate positive relationship between the data from IMU and the data from force platform was found in all cases.

## 4. Discussion

This study tested and verified a novel method utilizing the volume of convex polyhedron obtained by plotting three accelerations versus each other. It also proved the importance of incorporating the phase of the stance task on a foam surface with EC during the measurement process, since the results between patients with cerebellar ataxia and CG differed the most significantly in the CPVs observed during the FoS stance task, both in the EC and EO phases [40, 41]. This concludes that complicating stance tasks by reducing the mechanoreceptor perception highlights the differences in trunk movements between the CG and Pts. Also, the method identified significant differences between patients and CG in all instances of measurement. The CPV of the CG and Pts differed even in the hundreds of measured units of the CPV.

Although the method yielded results corresponding to those obtained by traditional methods in the case of Pts and CG standing on the FoS with EO and EC, the novel method revealed significant differences in the balance control between Pts and CG [8]. The above findings point to some complexity in the relation between the loss of perception and compensation for this loss by moving the trunk. In the case of the patients, the correlation coefficients indicate moderate and strong positive correlation between the CPV and ACE. The important information for the description of the situation is that very significant changes in the trunk position were seen only in the Pts. The reason for this is that the large movements only in the trunk, which is primarily used to improve the stability of the patient's body, have a great impact on changing the COM of the whole body and which corresponds to the position of the COP [42]. Conversely, in the case of the CG, the correlation coefficients indicate a weak or very weak correlation between the CPV and ACE. The reason for this is that the COP position is a result of a complex kinematic chain. Small movements of the COM of the trunk are overshadowed by movements of other body segments. Thus, in the case of the CG, small changes in trunk position may be different from the changes in position of the center of mass (COM) of the whole body, which differs from the COM of the trunk. Therefore, the lowest correlations correspond to the smallest movements of the torso when CG is standing with eyes open. The final reason why the standard parameter (e.g., ACE) is not correlated to CPV is because the CPV describes the complex 3D trunk accelerations (i.e., 3D movement) and the ACE describes distribution of only 2D data in transversal plane (2D space) and neglects movement in the third direction (i.e., vertical direction) [10]. Even a very small vertical movement which the posturography platform may fail to record can result in a significant change in the CPV. If we assume the area of CPV reflected into the horizontal plane, this may correlate with ACE. However if the reflected CP area is multiplied by a relatively small value of the vertical movement, which might be small compared to the horizontal movement, the volume of the resulting pattern varies according to the number of multiples in the value of vertical movement.

Significant correlations revealed between ACE and CPV during stance on FiS with EC suggest future use of IMU in the evaluation of postural stability in patients with, for example, cerebellar disease at smaller medical centers or even at home. Posturography platforms are spacious and less convenient for use in small medical centers or at home and IMUs might therefore replace the platforms in the therapy process, while enabling the patient to perform postural stability training individually with the IMU implemented in, for example, a mobile phone. Interpretation of the results from the measured data is identical to the interpretation of the results obtained by posturography platforms, and thus their use might be identical as well when it comes to therapy or evaluation of postural stability following surgeries. Based on the above it is clear that the determination and assessment of postural instability may be now additionally carried out by using the CPV, for example, in the form of examining 3D trunk acceleration. The major difference between utilizing triaxial IMU and the traditional method using 2D trajectory is the ability to describe the trunk movement in all three human body axes/planes. The novel method thus clearly found differences in postural control between Pts and CG and even greater differences in postural stability between Pts and CG. Clinical trials which are generally used to assess an impaired postural stability have large variability (e.g., Clinical Test of Sensory Interaction for Balance and others). This disadvantage could be eliminated using the proposed system and method which can be used to evaluate more subtle changes in postural stability in an individual patient course of treatment. Concluding from what is above mentioned, the potential of CPV may prove instrumental in gaining clearer insight into the postural stability and postural balance problems, which is crucial in medical examination and rehabilitation medicine.

This study also adopted a few limitations. The major one is that the size of the recruited subjects was rather smaller and possibly not fully applicable to the larger scope of population, even though it yielded statistically relevant results and it still might prove the results in a larger study. Another possible limitation is the chance of the existence of difference in comparisons which did not yield a statistically relevant result. However, it is safe to say that the sample of 10 Pts and 11 CG was relevant to design a preliminary study aimed on the study of degenerative cerebellar disorder sufferers using the basic features of the proposed techniques. Moreover, possible limitation might be also the nonhomogenous age groups that were compared. Even though previous works cited that age differences should not pose a significant influence on the presented results [38, 39], it would be interesting to use the presented method to compare age groups and thus verify or refute the influence of age on the relevance of the results.

## 5. Conclusion

All previous applications based on convex hull consider only two variables—two coordinates or two angles—not accelerations [22, 32]. However the three-dimensional version of the convex hull can also be used to study 3D movements of patients measured by triaxial IMU used in clinical practice. There are two main reasons for this design. The first reason is that one variable defining the shape of the 3D convex polyhedron allows us to study the change in the 3D movement as a whole by new and cheaper triaxial IMUs. Moreover, the combinations of the measurement of three accelerations during specific balance tasks could identify new and specific differences in balance control of patients compared to healthy subjects. The second reason for the measurement and evaluation of all three accelerations, instead of only two angles, is that the calibration of the cheap triaxial IMU may not be accurate and thus the measurement of accelerations in all three directions minimalizing the loss of information about the 3D movement as a whole. Particularly cheap IMUs in the contemporary mobile phones or watches, price of which is constantly falling and compared to expensive professional motion capture systems and posturography platforms is already considerably favorable, may find use in smaller medical centers as well as in long distance medicine thanks to the designed method. Also, inclusion of all three accelerations allows for minimalizing the influence of mispositioning of the sensor on a body segment. Thus, with the proposed method, it is capable of providing postural examination in everyday life using a cheap 3D IMU, and it is also possible to use the new method in a wide field of medicine including the rehabilitation with consideration of trunk coordination for physically challenged people.

The designed method and the 3D IMU not only are capable of replacing the expensive posturography platforms, but also they can become their complementary part. Posturography platforms allow for evaluation of body movement as a whole, and the introduced method provides for the evaluation of postural stability of a given segment. The proposed method of the evaluation of IMU data follows the traditional method used with posturography platforms and therefore is already familiar to the medical staff and easily interpreted. The use of parallel measurements of 3D data using IMU and posturography platforms for 2D data would require further research into the applicability of such solution and its contribution for medical examinations, for example, in rehabilitation process. For the future clinical use it would also be appropriate to add dynamic tests of postural stability to the static ones because of the larger complexity of the examination and also because performing the static tests during treatment does not correlate with the improvements of dynamic tests (which are also important for patient life quality).

## Figures and Tables

**Table 1:** Spearman's rank correlation coefficient between the volume of convex polyhedron and the area of the confidence ellipse of COP excursions of control group (CG) and patients (Pts) standing on a firm surface (FiS) and a foam surface (FoS) with eyes open (EO) and eyes closed (EC).

	CG EO	Pts EO	CG EC	Pts EC
FiS	0.03	0.53	0.37	0.93
FoS	0.03	0.83	0.18	0.70
